# Probabilistic performance assessment of complex energy process systems – The case of a self-sustained sanitation system

**DOI:** 10.1016/j.enconman.2018.02.046

**Published:** 2018-05-01

**Authors:** Athanasios Kolios, Ying Jiang, Tosin Somorin, Ayodeji Sowale, Aikaterini Anastasopoulou, Edward J. Anthony, Beatriz Fidalgo, Alison Parker, Ewan McAdam, Leon Williams, Matt Collins, Sean Tyrrel

**Affiliations:** School of Water, Energy and Environment, Cranfield University, MK43 0AL, UK

**Keywords:** Probabilistic performance assessment, Artificial neural network, Nano Membrane Toilet, Reinvent the Toilet Challenge, Energy recovery

## Abstract

A probabilistic modelling approach was developed and applied to investigate the energy and environmental performance of an innovative sanitation system, the “Nano-membrane Toilet” (NMT). The system treats human excreta via an advanced energy and water recovery island with the aim of addressing current and future sanitation demands. Due to the complex design and inherent characteristics of the system’s input material, there are a number of stochastic variables which may significantly affect the system’s performance. The non-intrusive probabilistic approach adopted in this study combines a finite number of deterministic thermodynamic process simulations with an artificial neural network (ANN) approximation model and Monte Carlo simulations (MCS) to assess the effect of system uncertainties on the predicted performance of the NMT system. The joint probability distributions of the process performance indicators suggest a Stirling Engine (SE) power output in the range of 61.5–73 W with a high confidence interval (CI) of 95%. In addition, there is high probability (with 95% CI) that the NMT system can achieve positive net power output between 15.8 and 35 W. A sensitivity study reveals the system power performance is mostly affected by SE heater temperature. Investigation into the environmental performance of the NMT design, including water recovery and CO_2_/NO_x_ emissions, suggests significant environmental benefits compared to conventional systems. Results of the probabilistic analysis can better inform future improvements on the system design and operational strategy and this probabilistic assessment framework can also be applied to similar complex engineering systems.

## Highlights

• A probabilistic model is developed to assess the performance of an NMT.
• Energy and environmental performance uncertainties of the system are qualified.
• A realistic prediction of the energy and environmental performance of the system.
• Probabilistic approach can be applied in other complex engineering systems.

## Nomenclature

φ: neuron output
θ: artificial neural network layer bias
y: artificial neural network output
f: neuron activation function
*ER*: equivalence ratio
$w_{i}$: artificial neural network node weight
$u_{i}$: stochastic variable
$e_{{\mathrm{NO}}_{x}}$: specific NO_x_ emissions (in mg NO_x_ kW h_e_^−1^)
$e_{{\mathrm{CO}}_{2}}$: specific CO_2_ emission (in mg CO_2_ kW h_e_^-1^)
${\overset{˙}{m}}_{{\mathrm{NO}}_{x}}$: rate of NO_x_ emission (kg s^−1^)
${\overset{˙}{m}}_{{\mathrm{CO}}_{2}}$: rate of CO_2_ emission (kg s^−1^)
${\overset{˙}{W}}_{\mathrm{net}}$: net power output of the integrated system

## 1 Introduction

In many developing countries, providing people with access to safe drinking water and hygienic sanitation facility is a key challenge to prevent the spread of infectious diseases. Globally, it is estimated around 2.4 billion people currently have no access to adequate sanitation facilities [1]. The conventional water flush toilet, widely available in the developed countries, is an inefficient use of water resources and requires intensive use of energy [2]. In addition, it requires public infrastructure including water supply, sewer and waste water treatment works, therefore is not feasible for low-income developing regions, including Sub-Saharan Africa. In densely populated urban areas, increasing the coverage of improved sanitation facilities is of particular urgency, due to the potential scale of disease outbreak. Sustainable ‘off-grid’ decentralised sanitation technologies are widely promoted by many international initiatives [3], as they are more suitable for regions with poor infrastructure. Sanitation systems such as rainwater-flushed-toilets, waterless urinals and composting toilets have been suggested in various studies as potential solutions to reduce or eliminate the use of potable water and improve rural health conditions [3], [4]. Despite the availability of such technologies, there remain significant technical and societal barriers which hinder the wide application of these sanitation technologies. Therefore, there are still strong technological and humanitarian incentives for the development of novel sanitation systems to improve the quality of life and disease control.

The ‘Reinvent the Toilet Challenge’ of the Bill and Melinda Gates Foundation is set to develop affordable, next-generation sanitary systems that can work without connection to external water, energy or sewerage systems [5]. The Nano-membrane toilet (NMT) project developed at Cranfield University provides an example of such an innovative solution for an off-grid, household-scale toilet that is able to treat human waste safely onsite [6]. The NMT unit is designed to operate without external energy and water supply under steady conditions. It integrates a membrane to recover clean water from urine and a compact energy conversion system to treat human faeces thermally. Recent studies confirmed that gasification and combustion are viable thermochemical technologies for the conversion of settled solids from human excreta into chemical or thermal energy [2], [7], [8]. Thus, the NMT has the potential to achieve self-sustained operation if energy recovery is optimised. Similarly to the development of any novel integrated process, process modelling is essential at the design stage to optimise the NMT system. Previous modelling efforts have focused on using thermodynamic equilibrium models [7], to simulate the thermochemical conversion of human faecal matter and to explore the thermodynamic viability of the NMT concept. Similar thermodynamic models have been applied widely to examine the conversion of various feedstocks including refinery sludge, sewage sludge and manure [9], [10], [11]. The traditional approach using deterministic data can reliably predict the system’s performance under any specified operating conditions and feedstock characteristics. This provides insights into complex processes and allows the identification of critical parameters and optimum operating conditions [12], [13]. A key limitation of such deterministic models is that they do not consider the effect of uncertainties of the input variables that are inherent in real life engineering systems, which are particularly relevant in the context of the NMT system, considering the highly stochastic properties of input variables (e.g. faeces and urine composition). Therefore, performance assessment using the deterministic approach provides an evaluation of the response of the system subject to fixed characteristic input values; however, it does not provide a definitive representation of the actual system’s performance that is often subjected to random fluctuations in the external and internal operating conditions [14].

**Fig. 1:**
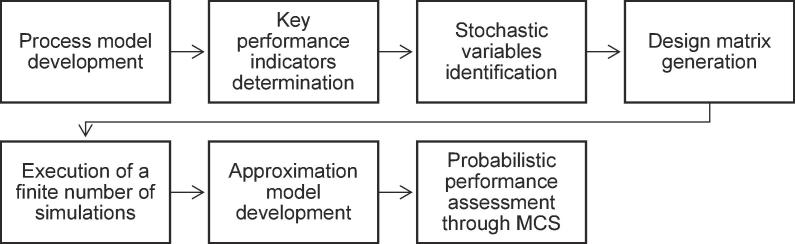
Overview of the probabilistic performance assessment framework.

This study presents the probabilistic thermodynamic performance assessment of the energy and water recovery system of the NMT based on an improved thermodynamic model of the NMT system. The system uncertainties arising from the stochastic characteristics of the input variables and their impact on the predicted performance of the NMT system were evaluated using an updated version of the probabilistic performance framework [15], [16] as shown in Fig. 1. This probabilistic modelling approach constitutes a non-intrusive formulation that sequentially combines a finite number of deterministic thermodynamic process simulations using artificial neural network (ANN) approximation models and Monte Carlo simulations (MCS) to map the response domain of the system under varying inputs. The outcomes of the analysis can enable a better interpretation of the system performance and support decisions for further optimisation from a design, operation and maintenance perspective. Novelty of this work is on the fact that the developed framework can be further applied in relevant complex engineering systems where uncertainties of inputs can significantly affect their performance and the use of ANNs can allow for confident evaluation of probabilities taking into account non-linear behaviour of performance indicators with respect to these uncertain inputs.

## 2 Methodology

### 2.1 Deterministic process & model description

**Fig. 2:**
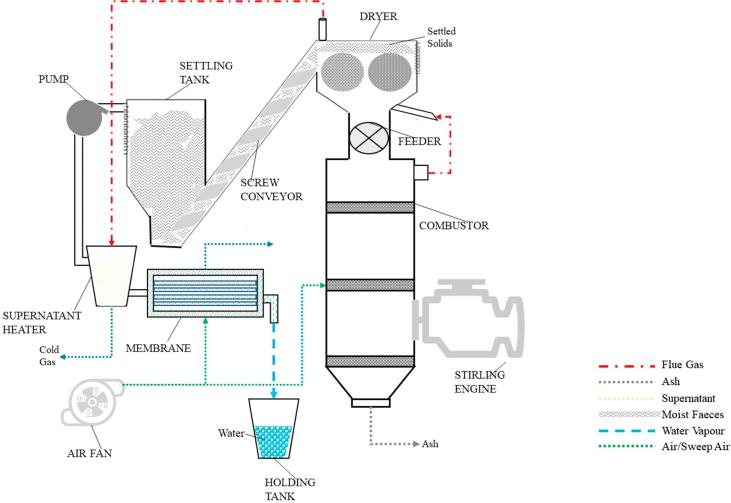
An optimised energy and water recovery system for the Nano Membrane Toilet.

The thermodynamic performance of the energy and water recovery system of the NMT unit is evaluated using a high fidelity deterministic process model, which is a revised version of the model described earlier by the authors [7]. All the processes both in the original and revised schemes were modelled in Aspen Plus simulation software (AspenTech Ltd., UK) using the thermodynamic equilibrium. The conceptual design of the NMT is shown in Fig. 2. Briefly, the urine, unbound water and partially-recovered bound water are first separated from human excreta by physical settlement to yield the supernatant and settled wet solids. The supernatant (primarily urine) is then purified by a hollow-fibre membrane to remove pathogens and odorous chemicals, whilst the settled wet solids are partially-dried in a dryer heated using the hot exhaust gas leaving the combustor, before entering a combustor for energy generation. This combustion process uses an excess of air to complete the conversion of the chemical energy in the settled solids to thermal energy. A SE is attached to the wall of the combustor (hot-end) and recovers thermal energy to generate electricity [17]. Membrane liquid-solid separation SE and the combustor enable the NMT unit to achieve its operational heat and power requirement, therefore maintaining a self-sustained operation.

Previous studies from this research group [2], [7] have considered the use of steady-state process modelling to describe the conceptual energy and water recovery systems of the NMT. The models described in these studies consider: (i) the faecal solids and resulting ash to be non-conventional streams, and the gases to have ideal behaviour; (ii) the solid transportation of the settled solids via a conveying and dewatering screw with specific power requirement of 200 J/kg_settledsolids_; (iii) the drying of the settled solids in a stoichiometric reactor coupled to a flash separator which receives the waste heat from the exhaust gas of the combustor; and, (iv) the thermochemical conversion of the partially-dried settled solids in a combustor using Yield and Gibbs minimisation-based reactors. Furthermore, the Pseudo-Stirling engine is considered to be an ideal cycle with an isentropic compressor and expander, and there is a supply of air from a compressor fan to cool the engine.

In this study, a number of modifications to the previous model were found to be necessary to achieve a more accurate simulation of real operational conditions. In the updated model, SE is modelled as a closed regenerative cycle, where the working fluid continuously operates within the expansion and compression spaces with net conversion of the heat energy to mechanical work. In addition, the compressor fan for an air supply to cool the SE was removed from the design to reduce system energy demand. To further improve the heat efficiency of the system, the heater component of the SE receives heat directly via conduction from the combustor wall rather than from the hot flue gas. As such, the heater and cooler components are modelled with HEATER blocks at temperatures of 600 °C and 50 °C respectively. The regenerator is modelled using a HeatX block with a hot/cold outlet temperature approach of 10 °C. The heater temperature is assumed to be the temperature of the working fluid and the maximum operating temperature of the SE. In addition, the combustor is modelled with a restricted temperature approach of 600 °C which ensures that the unit reaches the minimum temperature for the conversion of the partially-dried settled solids. To improve overall energy efficiency, 40% (on mass basis) of the exhaust flue gas is recycled back into the dryer to improve water removal in the settled solids. When leaving the dryer, the residual heat in the flue gas (∼60 °C) is used to preheat the supernatant to the temperature desirable for the hollow-fibre membrane that recovers water from the supernatant (illustrated as the red dotted line in Fig. 2). Similarly to the previous work, a simplified membrane water separation modelling approach was adopted as described by Hanak et al. [7] to account for the polypropylene membrane with a parallel flow configuration of a typical surface area of 0.5 m^2^.

An auxiliary energy requirement of 1643 J/kg_settledsolids_ is considered to accommodate the energy requirements for ignition, control and automation. A faeces generation rate of 0.21 kg/cap/day and urine generation rate of 1.5 dm^3^/cap/day are considered as the daily treatment requirement. These estimations are based on the range of values reported in [18], [19], [20], [21] and apply to a domestic-scale toilet in a ten-people household. The compositions of the settled solids and the supernatant are summarised in Table 1 and the detailed operational conditions of the considered system are listed in Table 2.

**Table 1:** Human excreta composition.

Settled Solids [2]		Supernatant [22]	
Component	Dry Basis (wt.%)	Component	As received (wt.%)
Proximate Analysis		Mass concentration	
Fixed carbon	0	Water	97.2
Volatile matter	82.6	Urea	1.38
Ash	17.4	Sodium chloride	0.82
Moisture (as received basis (wt.%))	77.0	Potassium chloride	0.17
	Dry Basis (wt.%)	Potassium sulphate	0.27
Ultimate analysis		Magnesium sulphate	0.08
Carbon	50.8	Magnesium carbonate	0.01
Hydrogen	6.8	Potassium bicarbonate	0.07
Oxygen	20.9	Lysine	0.01
Nitrogen	4.1	Asparagine	0.01
Ash	17.4	Phenol	0.03

**Table 2:** Initial design conditions for the revised energy and water recovery system.

Parameter	Value
Equivalent ratio (ER)	1.1
Specific power requirement for screw conveyor (J/kg_settledsolids_)	200
Auxiliary power requirement (J/kg_settledsolids_)	1643
Isentropic efficiency of air fan (%)	90.0
Mechanical efficiency of air fan (%)	99.8
Sweep Air Mass Flow (kg/day)	54.9
Combustor Restricted Approach Temperature (°C)	600
Dryer Temperature (°C)	105
Fraction of Exhaust Vented (%)	60
Exhaust Temperature (°C)	280
Desired moisture content of dried solids (wt.%)	20
Air preheater approach temperature (°C)	25
Settled solids per cap per day (g)	210
Supernatant per cap per day (dm^3^)	1.46
Supernatant outlet temperature (°C)	55
Stirling Engine Working Fluid Temperature/Heater Temperature (°C)	600
Stirling Engine Cooler Temperature (°C)	50

### 2.2 Probabilistic modelling approach

The probabilistic assessment framework developed in this study comprises seven definitive stages (Fig. 1). These stages are connected discretely, allowing for high fidelity tools to be employed and alleviating the need for an integrated probabilistic performance assessment model. Firstly, the process model of the system was developed in Aspen Plus, adopting modifications as described in Section 2.1. Secondly, key input variables and performance indicators of the process model were identified. Subsequently, stochastic variables in the process model and their statistical representation were assigned. Then, the approximation model was developed by mapping the response domain of the complex system based on the design matrix generated from the process model. Finally, a probabilistic performance assessment was conducted using a series of MCS to generate the joint probability density curves for the key process performance indicators. This was achieved by estimating the values of these indicators using the input dataset that contains one million sets that have been randomly generated according to the assigned distributions of the input variables. The selected resolution was determined sufficient following a sampling convergence study.

The process model indicates that there are 13 key input variables (Table 3) that influence the performance of the energy and water recovery systems of the NMT. A sensitivity study was conducted using a ±10% increase or decrease in the mean value of the key statistical parameters of the stochastic variables to determine their effect on the five key process performance indicators: SE power output, net system power output, water recovery performance, CO_2_ and NO_x_ emissions. The variables were assumed to be normally distributed since the distribution of the random variables was not known and the optimum specifications have not yet been identified. This generalisation of the stochastic variables using normal distribution is widely applied in the study of unknown real-valued random variables. In this probabilistic study, the distribution may not be the closest representation of the stochastic variables included, it is nevertheless the best available solution to gain more quantitative insights on the relationships between input and output variables. In addition, the probabilistic approach enables sensitivity analysis to be carried out to reveal the level of contribution of each stochastic variable to the system performance uncertainties. Therefore, more critical variables identified in a sensitivity analysis can be further studied in order to define them more accurately.

**Table 3:** Stochastic variables and their distribution.

Variable	Nominal value	Variation
Faeces per capita per day	210 g/cap/day	15%
Urine per capita per day	1.46 dm^3^/cap/day	15%
Equivalence Ratio (ER)	1.1	5%
Desired moisture content of dried solids	20%	10%
Stirling engine working fluid temperature	600 °C	5%
Stirling engine cooler temperature	50 °C	5%
Combustion Temperature	600 °C	10%
Preheated Air Supply	25 °C	10%
Faeces Ash/Volatile Matter Ratio	0.21	10%
Fraction of Exhaust Vented	60%	10%
Dryer Temperature	105 °C	10%
Exhaust Temperature	280 °C	10%
Sweep Air Mass Flow	54.86 kg/day	10%

Due to expected high uncertainties in operational parameters, a reliability approach based on MCS was used in this study to generate representative samples from stochastic distributions. There are other reliability methods including first and second order reliability methods (FORM/SORM); however, compared with MCS, these methods introduce one more stage of approximation leading to further uncertainties in the derived solution.

### 2.3 Stochastic response approximation model using artificial neural network

The ANN method was adopted to develop a robust approximation model that can provide the process input for probabilistic assessment. This method was selected, since stochastic analysis with direct simulations requires a large number of iterations and high computational effort – processes that could not be achieved directly in the Aspen Plus environment. For this reason, the study utilises the deterministic process model described in Section 2.1 to generate the design matrix, which in turn was used to develop the approximation model. Such a model links the process input variables to the output variables, the latter of which can be considered as input variables to the deterministic model.

ANN is inspired by the structure of biological neural networks and the process they utilise to solve problems [23]. As opposed to the conventional approximation models, such as surrogate or response surface modelling, ANN ‘learns’ the relationships between the inputs and outputs by training [24]. It is also known to be able to reliably represent multiple outputs considering multiple inputs [25], even if the system’s behaviour is highly non-linear [24]. The most common structure of the ANN comprises an input layer, one hidden layer with sigmoid neurons, and an output layer with linear neurons [24], [26]. The input to each neuron can be the network input from the input layer, the output of the neuron in the previous layer, and an externally applied bias [23]. The output of each neuron is the function of the weighted sum of the neuron inputs, with the hyperbolic tangent sigmoid transfer function (Eq. (1)) used in the hidden layer and the linear function (Eq. (2)) used in the output layer. The weights and bias are determined in the training process by minimising the error between the ANN outputs and the design matrix.

(1) $$f(\varphi )=\frac{2}{1+{\text{e}}^{-2(\overset{k}{\underset{i=1}{\sum }}w_{i}\cdot u_{i}+\theta )}}-1$$

(2) $$f(\varphi )=\overset{k}{\underset{i=1}{\sum }}w_{i}\cdot u_{i}+\theta$$

where φ is the Neuron output; θ is the ANN layer bias; $w_{i}$ is the ANN node weight and $u_{i}$ is the stochastic variable.

Using the MATLAB Neural Network Fitting toolbox, a two-layer feed-forward ANN with ten sigmoid hidden neurons and linear output neurons was developed to map the system response generated from the process model (based on the design matrix inputs). To ensure an accurate prediction by the ANN, the data in the design matrix were divided between training (70%), validation (15%) and testing (15%) samples. Neural network training was performed to adjust the weights of all the connecting nodes until the desired network performance was reached. The evaluation of network performance is essentially a non-linear optimisation process and the objective function involves minimisation of an error function, e.g. mean squared error (MSE). In this study, the Bayesian regularisation training algorithm was used to obtain the optimal values of the adjustable parameters, weights and biases. The MSE performance function (Eq. 3) was used to assess the network performance.

(3) $$\mathrm{MSE}=\frac{1}{N}\overset{N}{\underset{i=1}{\sum }}{\left(z_{i}-y_{i}\right)}^{2}\to \min$$

where, z_i_ = the targets, y_i_ = network outputs and N = data size.

### 2.4 Performance assessment

The design goal of the NMT unit is to generate a sufficient amount of electricity for the entire system to become self-sustained and this is in addition to the need for maximum water recovery efficiency from the system. As such, the key performance indicators considered in this study for the deterministic and probabilistic process models are: (i) power output of the SE (*W*_S_), which indicates the mechanical work recovered from the waste heat; (ii) net power output of the entire system (*W_net_*), which indicates any excess power available after the systems’ power requirements are satisfied; (iii) water recovery efficiency (η_WRE_); and (iv) net emissions including CO_2_ and NO_x_ (represented as NO_2_ in the ASPEN model). The indicators were obtained using Eqs. (4-7).

(4) $$W_{\mathrm{net}}=W_{S}-[W_{\text{DRYER}}-W_{\text{CRUSHER}}-W_{\text{SWPAIR}}-W_{\text{feeder}}-W_{\text{URPUMP}}-W_{\text{AIRFAN}}-W_{\text{MEMB}}-W_{\text{AUX}}]$$

(5) $$\eta_{\text{WRE}}=\frac{\text{Moisture Output}}{\text{Moisture Input}}*100=\frac{{\overset{˙}{m}}_{\text{MEMB}}*(1-{\overset{˙}{v}}_{\text{MEMB}})+{\overset{˙}{m}}_{\text{URHEATER}}*(1-{\overset{˙}{v}}_{\text{URHEATER}})}{{\overset{˙}{m}}_{\text{URINE}+[{\overset{˙}{m}}_{\text{FAECES}}*{\overset{˙}{m}}_{C}]}}*100$$

(6) $$e_{{\mathrm{CO}}_{2}}=\frac{{\overset{˙}{\overset{˙}{m}}}_{{\mathrm{CO}}_{2}}}{W\text{net}}$$

(7) $$e_{{\mathrm{NO}}_{x}}=\frac{{\overset{˙}{\overset{˙}{m}}}_{{\mathrm{NO}}_{x}}}{W\text{net}}$$

where W_DRYER_, W_CRUSHER_, W_SWPAIR_, W_FEEDER_, W_URPUMP_, W_AIRFAN_, W_MEMB_, W_AUX_ are the power requirement (W) for the dryer, crusher, sweep-air pump, settled solids feeder, urine pump, compressor air fan, membrane and auxiliary components respectively. $\overset{˙}{m}\;\text{MEMB and}\;\overset{˙}{m}\;\text{URHEATER}$ represent the mass flow of membrane outlet and supernatant heater respectively, while $v\text{MEMB and}\;\overset{˙}{v}\;\text{URHEATER}$ refer to the vapour fraction of the membrane outlet and supernatant heater respectively. $\overset{˙}{m}\;\text{URINE and}\;\overset{˙}{m}\text{FAECES}$ represent the mass flow of water in urine and faeces respectively. $\overset{˙}{m}C$ is the moisture content of the faeces (wt.%). $e_{{\mathrm{CO}}_{2}}\;\text{and}\;e_{{\mathrm{NO}}_{x}}$ correspond to specific emissions, while ${\overset{˙}{m}}_{{\mathrm{CO}}_{2}}\;\text{and}\;{\overset{˙}{m}}_{{\mathrm{NO}}_{x}}$ are the net emissions in g/hour for CO_2_ and NO_x_ respectively.

## 3 Results

### 3.1 Deterministic performance assessment

The performance assessment of the conceptual energy and water recovery system of the NMT from the deterministic process simulation, is presented in Table 4.

**Table 4:** Deterministic performance indicators of the conceptual energy and water recovery systems of the NMT.

Indicator	Value
Adiabatic Flame Temperature (^o^C)	1367.2
Exhaust Gas Temperature (^o^C)	280
Dryer Temperature (^o^C)	105
Stirling Engine Power Output (W)	65.8
Stirling Engine Power Consumption (W)	397
Stirling Engine Efficiency (%)	16.8
Dryer Heat Requirement (W)	41.2
NMT Net Power Output (W)	22.3
NMT Heat Input (W)	88.8
NMT Net Efficiency (%)	25.1
Water Recovery Efficiency (%)	74.1
Total CO_2_ and CO emissions (kg/kg_settled solids_)	0.42
Total NO_x_ (kg/kg_settled solids_)	0.02

The results show that the main energy-intensive component of the NMT system is the dryer, which requires 41.2 W, i.e. ∼62% of the SE power output. This value is the energy required to reduce the moisture content of the faeces from 77 to 20 wt% (as received basis). Other system components for transportation, ignition, control and automation require ∼2.3 Wh/kg_settled solids_. As such, the net efficiency of the NMT is deduced to be ∼25% at net power output of 22.3 W per hour of operating the system. In this case, the equilibrium model assumes that the fuel is completely converted to energy, and the combustor reaches an adiabatic flame temperature of 1367.2 °C under a restricted temperature approach of 600 °C.

Furthermore, the results show that the power consumption of the SE is ∼397 W per hour of operation. This energy accounts for the heat recovered from the combustor and regenerated from the cyclic expansion and compression processes. Thus, the SE power output of 65.8 W places the SE efficiency at 16.8%, a value that is within the range reported in [27], [28], [29]. In terms of net emissions, the system produces 0.42 kg of CO_2_ emissions and 0.02 kg of NO_x_ emissions per kg of settled solids. This corresponds to specific CO_2_ emissions of 1.66 mg/kW h and specific NO_x_ emissions of 0.08 mg/kW h. Canova et al. [30] reviewed the emission data for various technologies, including fuel-lean burn systems, and reported some specific NO_x_ emissions of 650–1500 mg/kW h_e_ for large electric power systems and 208 mg/kW h_e_ for boilers of different sizes and types. Goyal et al. [31] reported the specific CO_2_ emission index for a micro-capacity single cylinder diesel engine at fuel load to be 0.59 kg/kW h_e_ and 0.48 kg/kW h_e_ under single generation and combined heat and power mode respectively. Khatri et al. [32] on the other hand showed that the emission rates varied from 0.12 to 0.19 kg/kW h (full to no load case) in a micro-trigeneration system. The outcomes in this study are thus comparable to those of conventional fuels and systems.

The above deterministic assessment and those published elsewhere [7] predict that high water recovery efficiency and positive energy gain can be achieved from the NMT system. However, these outcomes demonstrate the key design targets for the self-sustainable operation of the NMT and provide the system’s performance under specified operating conditions. The analysis excludes the stochastic characteristics of the input variables and offers no information on the probability of achieving this performance outcome (i.e. system reliability). For instance, key variables such as settled solids and supernatant per capita per day, as listed in Table 3, have a wide range of reported values [2], [33], [34]. Rose et al. [19] report, on average, a healthy individual generates faecal wet mass values in the range of 51–796 g/day. When individual variation is accounted for, the range extends to 15–1505 g/cap/day. These generation rates varied in terms of age, diet, body weight, average daily food intake and country (as subject to economic and health status). The study also reports a range of urine generation rates of 0.6–2.6 dm^3^/cap/day. Such input variations can affect the probabilities of achieving a specific performance indicator and cause uncertainty in the overall performance of the NMT. This subsequently can affect the unit’s commercialisation and market uptake. Therefore, uncertainties that reside within the NMT unit must be appraised alongside its environmental impacts in order to establish the wider application of the technology. A quantitative probabilistic analysis allows the further understanding of how stochastic variables influence the outcome of the performance model by predicting the operating envelopes of the systems’ parameters under uncertain input conditions. The output of the analysis can inform future improvements on the unit, both with regard to its design and its operational strategy.

### 3.2 Neural network performance training results

**Fig. 3:**
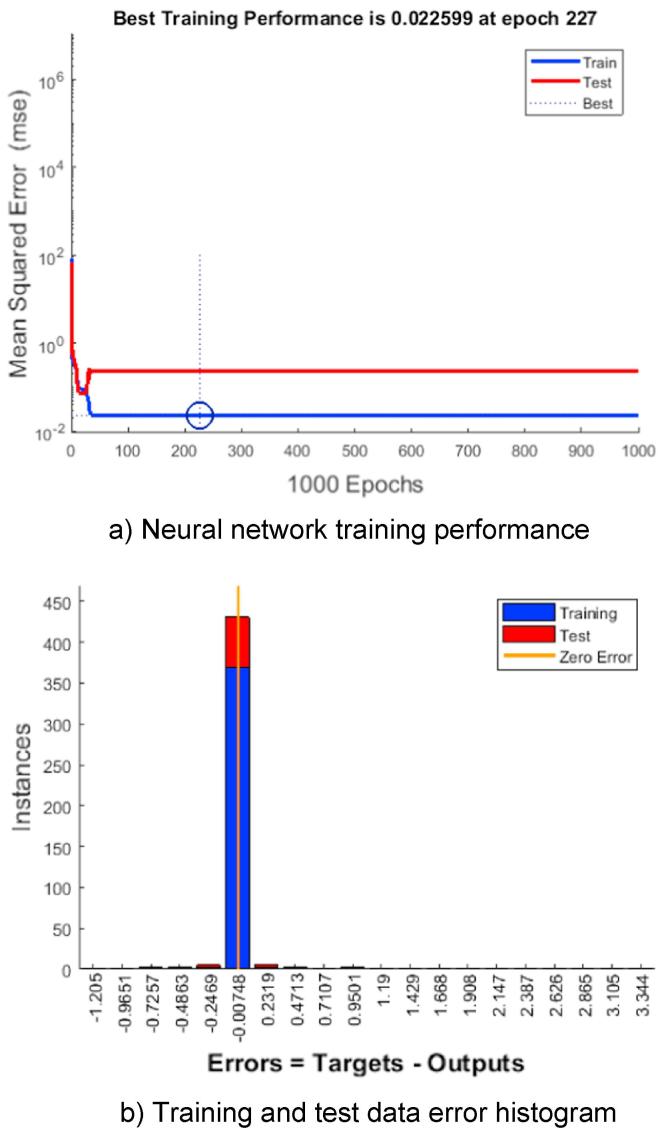
ANN training performance.

Fig. 3a exhibits the training, validation and test errors. Clearly the final mean-square error is small, and the test set error and validation set error have similar characteristics, indicating a model of high predictive quality.

Training was stopped at the lowest MSE for the validation set before the MSE started to increase (Fig. 3a), therefore there is no indication of the onset of overfitting. Typically, during the training process the error decreases indefinitely with the increasing number of hidden nodes or training cycles, as shown in Fig. 3a. However, a subsequently slowed reduction in error was observed. This is attributed to the excessively large number of training cycles due to network memorisation, in addition to the use of a large number of hidden nodes causing overfitting. The final (optimal) neural network architecture is obtained at the onset of the increase in test data error [35]. In addition, as shown in Fig. 3b, the majority of the training and test data are close to the zero-error baseline; therefore, there is no indication of significant data outliers, which are data points where the fit is significantly worse than the majority of the data.

### 3.3 Probabilistic performance assessment

#### 3.3.1 Net & stirling engine power output

**Fig. 4:**
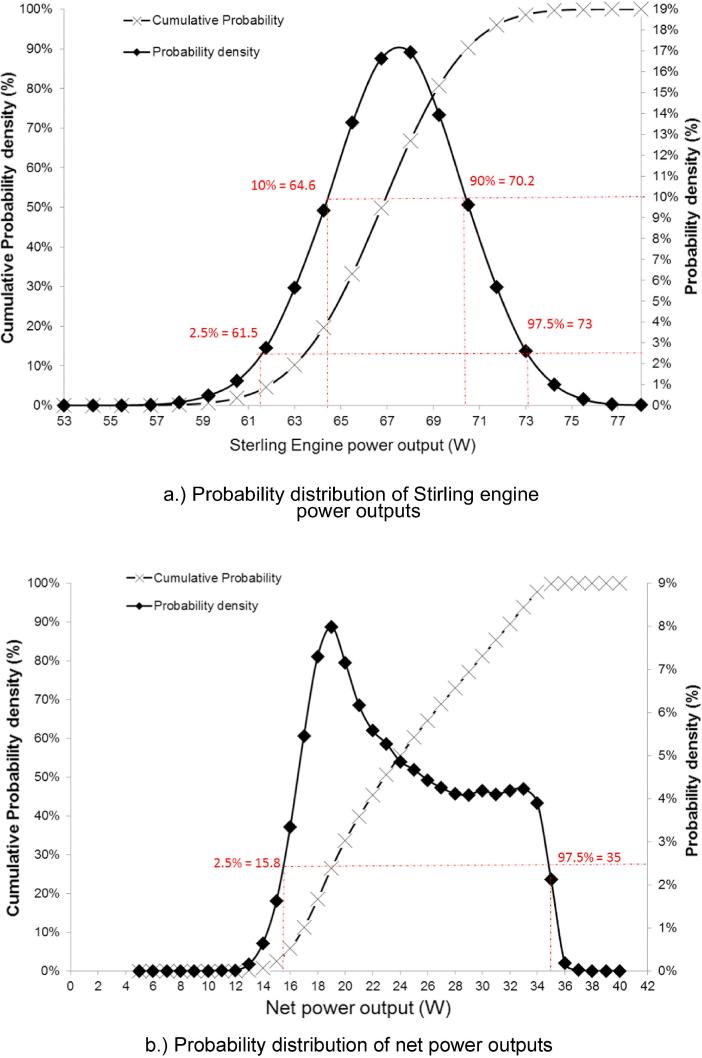
Assessment of thermodynamic performance.

The joint probability density functions of both net system and SE power outputs are plotted in Fig. 4a and b. The results show negatively-skewed normal distributions, indicating non-linear correlations between power outputs and the stochastic parameters. The skewness of both distributions strongly suggests high probabilities of achieving higher power outputs. Thus the probability plot in Fig. 4a suggests that the SE can achieve a positive power output in the range of 61.5–73 W at a 95% confidence interval (CI). A high probability is also observed with 80% CI that the engine can reach a reasonable power performance between 64.6 and 70 W.

The probability plot of net system power output (Fig. 4b) shows there is a high probability at a 95% CI for the NMT to achieve positive net power output within the range of 15.8–35 W. Previous deterministic study [7] estimated a value of 1.9 W for net system power output; however, the probability analysis carried out in this study indicates that the energy performance of the NMT, as previously estimated using the deterministic method, was conservative. Compared to deterministic analysis, it is clear that the probabilistic results provide a more meaningful interpretation of the system performance and approve with high confidence the NMT design concept to be energetically self-sustained, despite the uncertainties inherent in the system.

#### 3.3.2 Water recovery performance

**Fig. 5:**
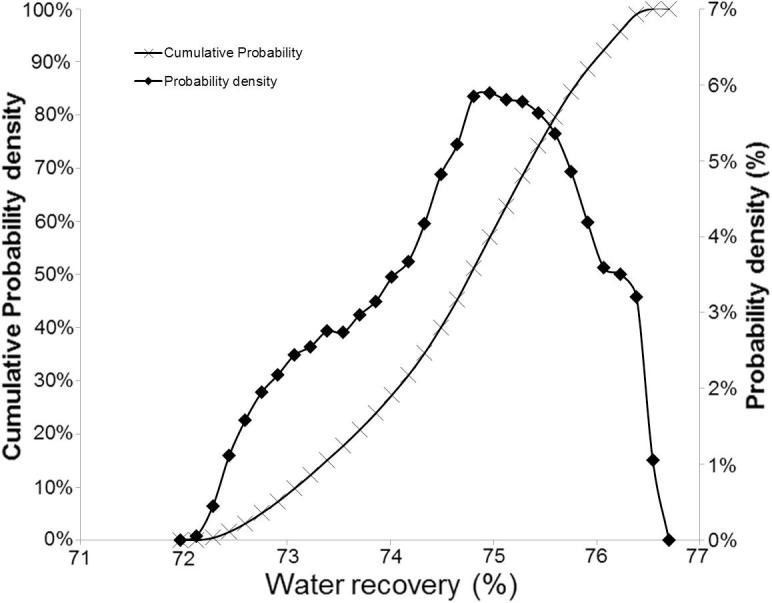
Probability distribution of water recovery efficiency.

The use of membrane technology in the NMT unit enables it to achieve a high percentage of clean water recovery from urine and faecal matter. The recovered water can be recycled for irrigation, cleaning etc., thus providing additional environmental and socio-economic benefits for regions with severe water shortages. The cumulative probability in Fig. 5 shows conclusively (at ∼ 100% probability) that across the whole operational envelope and considering all stochastic variables, the system consistently produced a high percentage of water recovery between 72 and 76.6%, offering considerable environmental benefit in water saving.

#### 3.3.3 CO_2_ and NO_x_ emission assessment

Similarly to the combustion of any fuel, there are environmental concerns associated with emissions when burning human faeces. In this study, environmental emissions include CO_2_ and NO_x_ because of the content of C and N in the initial faecal matter. As such, it yields its carbon and nitrogen under oxidation to produce CO_2_ and NO_x_ (particularly at high temperatures).

**Fig. 6:**
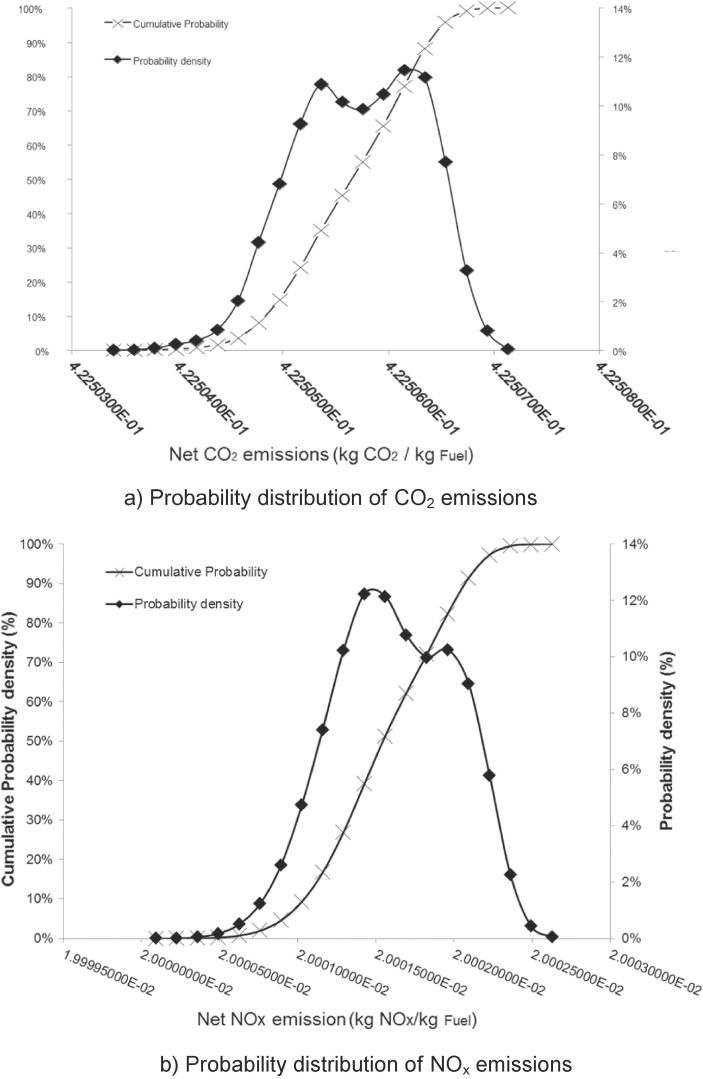
Probability distribution of net emissions.

The probabilistic analysis suggests that CO_2_ and NO_x_ emissions from the combustion of the faecal matter are not affected by the stochasticity of the input variables. Net specific CO_2_ and NO_x_ emissions (per kg of settled faecal matter) are found at 0.42 kg and 0.02 kg respectively with extremely low variances (Fig. 6). This is expected due to the assumption of the complete combustion of faecal matter. As assumed in this study, an NMT unit receives 2 kg of settled faecal matter per day in a ten-people household; the system will, therefore, produce 0.82 kg CO_2_ and 0.04 kg NO_x_ per day. This is equivalent to ∼306 kg CO_2_ and 14.6 kg NO_x_ emissions per year.

Compared with a conventional flush toilet, the waterless design of the NMT also considerably reduces the CO_2_ emissions associated with potable water supply and wastewater treatment. A typical flush toilet on average uses 36 L of water per person per day [36]; therefore for a ten people household, the annual water saved from using NMT would be 131,400 L (36 L × 10 people × 365 days) In addition, this results in the discharge of the same amount of waste water into the sewerage system. Considering the UK figures for potable and waste water, the associated CO_2_ emissions are 0.452 and 0.781 g litre^−1^, respectively [37], the CO_2_ emissions saved from flush water would be at ∼160 kg CO_2_ annually.

## 4 Sensitivity analysis and operational implications

**Fig. 7:**
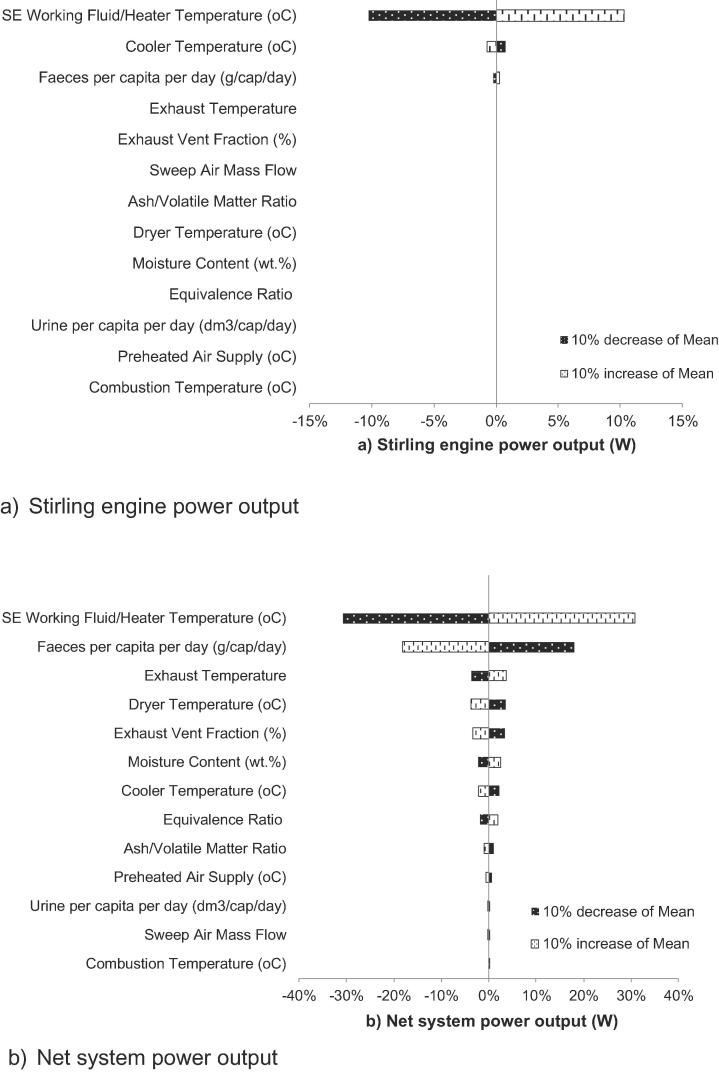
Percentage contributions of the input variables to power outputs.

**Fig. 8:**
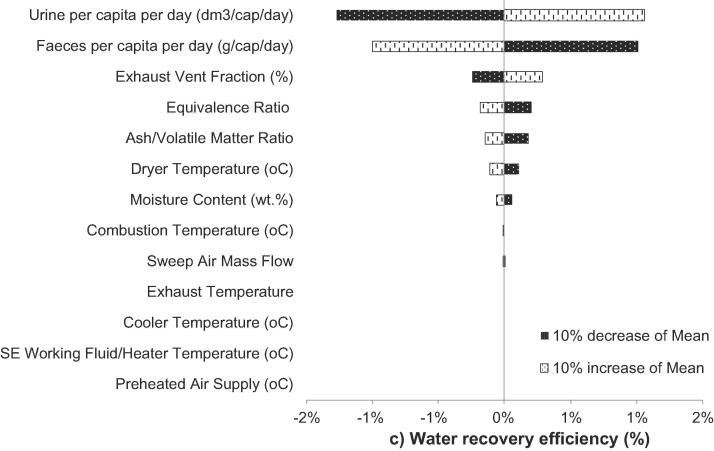
Percentage contributions of the input variables to water recovery efficiency.

**Fig. 9:**
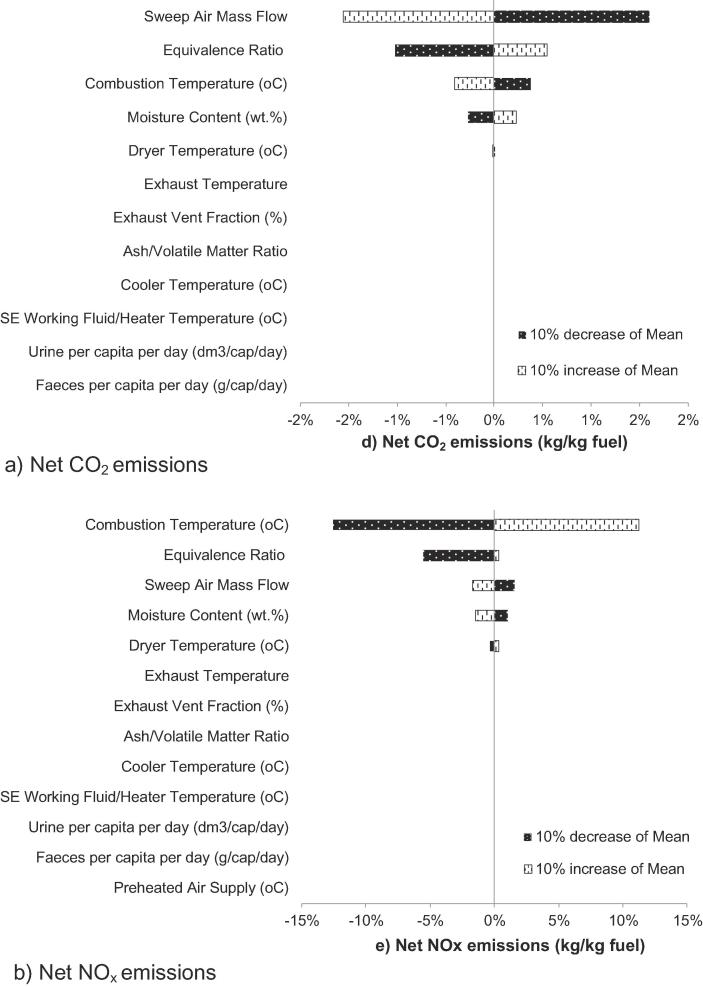
Percentage contributions of the input variables to emissions.

The sensitivity analysis is based on an assessment of an increase or decrease of 10% in the mean value of the key statistic parameters of the stochastic variables and reveals the varying impact that the input variables have on the process performance indicators. The outcomes of the sensitivity analysis are presented in Tornado plots in Fig. 7, Fig. 8, Fig. 9.

Fig. 7a and b show that the SE heater temperature, and hence the working fluid temperature, is the most significant variable to affect the SE and net system power outputs. It was observed that a 10% increase in the heater temperature increased the SE power output by ∼10% and conversely. The magnitude of the effect was considerably larger on the net system power output, at around 31%. These results were expected because the maximum temperature of the SE is at the heater, i.e. the hot-side of the engine connected to the combustor wall, and consequently any waste heat not recovered from the combustor is lost to the environment and contributes to heat transfer losses. By contrast, an increased heat input at this point indicates the potential to recover more energy for the system. The result also shows that a 10% increase in the cooler temperature decreases both the engine and net system power outputs, though to a lesser degree (∼1% for SE power output and ∼2% for net system power output). This is due to a reduced capacity of the working fluid to recover waste heat from the system.

Since the SE is the key component of the unit that converts heat energy into mechanical work, it is essential to pay attention to the design and operational factors that can affect the engine’s efficiency. Various factors can be considered to improve the performance and efficiency of the SE, such as having a low total dead volume ratio, high heat transfer rate, and low pressure and work losses, increasing the heat source temperature and decreasing the heat sink temperature, or operating at an optimum frequency. Thus, the dead volume ratio is reduced by reducing the clearance between the expansion and compression spaces. A continuous cyclic operation with consistent heat transfer and regeneration reduces pressure and work losses. Improving the temperature distribution in the hot expanding and cold compression spaces enhances the heat transfer rates and decreases thermal resistance [38].

Further improvements in system performance can be achieved using structural dynamic analysis. The heat transfer rate via the heater, cooler and regenerator can be improved with the use of materials and fluids with relatively high thermal conductivity. The effectiveness of the heat exchangers can be improved using fin structures to increase the surface area and improve the heat transfer rate. Precision engineering of the seal clearance can prevent leaks of the working fluid, depending on engine configuration. This working fluid can vary from air to inert gases, and operates across the three heat exchangers of the SE (i.e. heater, regenerator, and cooler) at different temperatures and occupying variable volumes. Low density gases, such as hydrogen and helium, are more suitable for SE working fluid due to their superior heat diffusion performance compared to air. It is therefore noted that the properties of the working fluid can directly affect the performance of the SE, hence the net system power output.

Other operational variables found to affect net power system outputs include dryer and exhaust gas temperatures and fraction of exhaust vent (FEV) due to their implications for the energy requirements and efficiency of the dryer. In this study, it is observed that a 10% decrease of the exhaust gas temperature can result in a ∼4% decrease of net system power output, whilst a 10% decrease of dryer temperature or 10% reduced FEV both result in ∼3–4% increase of system power outputs due to reduced energy requirements from the system.

The urine and faeces generation rates are the key stochastic variables affecting the water recovery efficiency, although their effect is limited (∼1–2%) as shown in Fig. 8. Since the supernatant (mainly urine) and faeces contain high water contents, increasing the input of faeces and urine increases water output. Other variables that affect water recovery efficiency include FEV, equivalence ratio (ER), ash/volatile matter ratio (A/V) and dryer temperature. However, a ±10% change of these variables results in efficiency changes lower than 1%.

Net CO_2_ emissions are most significantly affected by four key stochastic variables: sweep air mass flow, equivalence ratio (ER, on air-fuel basis), combustion temperature and the moisture content of the faecal material (Fig. 9a). All four variables have a known impact on the formation of combustion products. In this study, it is observed that reducing the ER value can lead to a more significant reduction of net CO_2_ emissions, whilst CO_2_ emissions are less affected by increasing the ER. This is because the base-scenario is assumed for the system to operate under a slightly fuel-lean condition (ER = 1.1) to achieve more complete combustion. The decrease in ER reduces the degree of combustion which leads to lower combustion temperature and an undesirable progressive production of CO with other unburned hydrocarbon emissions.

In Fig. 9b, the results show that net NO_x_ emissions increase with an increased combustion temperature or ER. These results are expected because the production of nitrogen oxides increases at higher temperatures [39] and under increased oxidation conditions related to larger air-fuel ratios (i.e. larger ER). At a lower ER, net NO_x_ emissions are significantly reduced because they are produced at lower combustion temperatures. This indicates that an improved emission performance is possible for the NMT if appropriate combustor design and operational conditions are in place. This includes the engineering design of the air nozzles, combustion zone and stages, and ignition system and a proper understanding of the nature and properties of the faecal material.

This paper has focused on the assessment of the steady-state probabilistic performance of the NMT system, and the results reported have highlighted the key contributing variables that influence the selected performance indicators. Variations in the design and operation can be implemented based on these results in order to achieve more favourable operational outcomes, i.e. more positive net power output and water recovery levels, or a reduced variation in the joint probability density, i.e. a more consistent operation.

Although the analysis in this work is based on steady-state operation, it should be noted that the system performance is expected to vary during its transient state or as a result of mechanical degradation. The latter may be due to various time dependent failure modes/mechanisms, such as membrane fouling, or increase of losses in the system, eventually denoting failure of the unit to fulfil its intended function. This will occur as a result of the gradual reduction of the mechanical resistance of the system and at the same time the increase in load effects which will in sequence denote an increase of the probability of failure of the system. The framework that has been developed and reported in this paper can be further applied to account for this performance deterioration, considering a time-dependent reliability assessment. This assessment would quantify the performance deterioration of the system over time, which in turn would allow for efficient material selection, specification of the operational envelope and requirements for maintenance of key components, reducing downtime of the system and increasing operational availability. This could be achieved through maximisation of the utilisation factor of different components, intervening just before functional failure occurs.

## 5 Conclusions

This study demonstrated the feasibility of a novel design of an NMT powered by energy produced from human faeces. Using a deterministic process model in combination with an advanced quantitative probabilistic assessment approach, the effect of system uncertainties on the predicted NMT unit performance (i.e. thermochemical energy conversion into power, water recovery and CO_2_ and NO_x_ emissions) was determined.

In thermodynamic performance assessment, probabilistic analysis suggests SE power output can achieve a value in the range of 61.5–73 W with 95% CI. In addition, there is high probability (with 95% CI) that an NMT unit can achieve positive net power output between 15.8 and 35 W. Sensitivity studies reveal the system power performance is mostly affected by SE working fluid and heater temperature. The probabilistic analysis shows the current NMT design can achieve 72–76.6% water recovery across the whole operational envelope. Sensitivity analysis revealed water recovery was most dependent on the daily loading of urine and faecal feedstock, exhaust vent fraction and ER. Emission performance assessment suggests CO_2_ and NO_x_ emissions are not affected by stochastic variables. Net CO_2_ and NO_x_ emissions of NMT were found at 0.42 kg and 0.02 kg per kg of settled wet faecal matter respectively.

The results from this probabilistic study validate the overall NMT design and provide profound insights into future system optimisation. Results of the analysis can better inform future improvements on the system both with regard to its design and its operational strategy. Further, the framework developed can be applied for similar complex, non-linear systems, where performance is highly affected by the stochasticity of inputs.
